# A reference guide for tree analysis and visualization

**DOI:** 10.1186/1756-0381-3-1

**Published:** 2010-02-22

**Authors:** Georgios A Pavlopoulos, Theodoros G Soldatos, Adriano Barbosa-Silva, Reinhard Schneider

**Affiliations:** 1 Structural and Computational Biology Unit, EMBL, Meyerhofstrasse 1, Heidelberg, Germany; 2 Computational Biology and Data Mining Group, Max-Delbrück Center for Molecular Medicine, Robert-Rössle-Strasse, 10, D-13125, Berlin, Germany

## Abstract

The quantities of data obtained by the new high-throughput technologies, such as microarrays or ChIP-Chip arrays, and the large-scale OMICS-approaches, such as genomics, proteomics and transcriptomics, are becoming vast. Sequencing technologies become cheaper and easier to use and, thus, large-scale evolutionary studies towards the origins of life for all species and their evolution becomes more and more challenging. Databases holding information about how data are related and how they are hierarchically organized expand rapidly. Clustering analysis is becoming more and more difficult to be applied on very large amounts of data since the results of these algorithms cannot be efficiently visualized. Most of the available visualization tools that are able to represent such hierarchies, project data in 2D and are lacking often the necessary user friendliness and interactivity. For example, the current phylogenetic tree visualization tools are not able to display easy to understand large scale trees with more than a few thousand nodes. In this study, we review tools that are currently available for the visualization of biological trees and analysis, mainly developed during the last decade. We describe the uniform and standard computer readable formats to represent tree hierarchies and we comment on the functionality and the limitations of these tools. We also discuss on how these tools can be developed further and should become integrated with various data sources. Here we focus on freely available software that offers to the users various tree-representation methodologies for biological data analysis.

## Introduction

Tree data structures and representations are essential in biological studies. They are able to show hierarchical organizations of biological data and concepts; for example, some of the most well known efforts for hierarchical representations are the Gene Ontology (GO) [1] that describes the functional annotation of genes via a hierarchically organized set of terms and phrases and the Unified Medical Language System (UMLS) [2] that has a biomedical focus as discussed later. A prime example of tree representations is the so-called tree of life [3] which displays evolutionary relationships between species and how they separated and evolved over time. Tree representations are also valuable for classification and clustering visualization of biological data.

Evolutionary studies were always a very important field of biological research. Currently, the modern sequencing techniques and their improvements make it easy to sequence and analyze more and more species. There are approximately 1.7 million identified species, which is just a fraction of the total number of species that exist. Only about 80,000 of these species have been analyzed for evolutionary relationships and have been assigned into a hierarchy [4]. The major challenge remains: the creation of the biggest possible phylogenetic tree of life that will classify all species showing their detailed evolutionary relationships. Ideally, all of the species recognized thus far should have a place in that phylogenetic tree. Therefore, proper visualization tools that will be able to display very wide and deep hierarchies are necessary.

Chip-Chip arrays, microarrays, and other proteomics or trascriptomics technologies improve every day and the data produced by them often require statistical and clustering analysis [5], the results of which are usually visualized by tree hierarchies. Nevertheless, methods that greatly simplify the analysis and interpretation of biological data are not enough. Well-designed visualization applications that are developed, eventually transform raw data into logically structured and visually tangible representations. Their main purpose is to reveal those patterns and structures that remain hidden in the raw data and are not obvious to perceive. Unfortunately, nowadays, the current visualization tools are unable to efficiently visualize vast amounts of data in tree hierarchies and the big challenge remains: to handle the overload of information and make it easier to understand and explore.

In this review, we summarize and evaluate tree visualization tools that have been developed to analyze and visualize biological relationships. There is a wide variety of tree visualization tools available, which makes an exhaustive search of all of them impossible. Therefore, we focus on the most recent visualization tools produced in recent years and on those widely used. Initially, a formal definition of trees as graphs is given together with the most common tree types, representations and layout algorithms. Next, we present the widely used standard and uniform file formats that are able to describe tree hierarchies in computer-readable raw text format. We continue with a brief description of major biology research fields for which tree representations are important and explain the reason for that. A survey on some of the best known visualization tools follows. Taking into account that each tool comes with different properties, functionalities, advantages and disadvantages, we try to evaluate and comment on their strengths and weaknesses, as our purpose is not to compare but to aid researchers in choosing the most suitable visualization tool for their studies. Finally, we present software, tools and packages or libraries that can serve to perform analysis and manipulation of data, which can be presented with tree structures. In conclusion, we discuss future directions and how next generation tree viewers can be more efficient to handle the upcoming vast amounts of biological data.

## Tree definitions

In this paragraph, formal descriptions are provided, related to the tree data structures of interest. Simple definitions and terminologies are presented with the purpose to introduce the tree structure concept; an exhaustive description is not in scope.

### Terminology

A *tree *is a connected graph G = (V, E) that does not contain cycles, where V and E represent the vertices and the edges of G, respectively. This means that any two nodes of a tree are connected via a single path and that there is no link that can be traversed more than once. For every tree applies that |E| = |V|-1, where |E| is the number of connections and |V| is the number of nodes. In a tree, each node may have one or more children but only one ancestor. In the case of a *binary tree *each node has maximally two children. The nodes may correspond to events of divergence, which is most commonly the case in phylogenetic and clustering analyses. *Root *of a tree is the highest ancestor of the hierarchy whereas *leaves *are the nodes that have no children. As *internal *or *inner *is defined a node that is not a leaf and has children. A *subtree *is a fraction of the graph G, the hierarchy of which can stand as a complete tree by itself. Every node of a tree can be a root node to form a subtree. The *height *of a node is defined as the length, i.e. the number of edges, from the node to the longest downward, i.e. away from the root, path to a leaf. The height of the tree is defined by the height of the root. Correspondingly, the *depth *of a node is the length of the path to its root. There are trees, however, for which there is no natural orientation and usually there is no node defined as root; these trees are called *unrooted *trees. Consequently, trees can be classified as *rooted *or *unrooted *depending on the presence of a root node at the top of the hierarchy, or not, respectively. While unrooted trees can always be generated from rooted ones, the opposite does not apply; a rooted tree cannot always be reconstructed from an unrooted one.

A special category of trees, due to the biological interest in displaying and studying evolutionary relationships among species, are the so called *phylogenetic trees*. A phylogenetic tree (T, t) is parameterized by a topology T, i.e. simply the set of edges, and a mathematical vector t that represents the edge lengths. A rooted phylogenetic tree is a directed tree with a unique node that is in the highest part of the hierarchy and is recognized as the root node of the tree. Unrooted phylogenetic trees illustrate the relatedness of the leaf nodes without making assumptions about common ancestry.

### Tree representations and layouts

**Figure 1:**
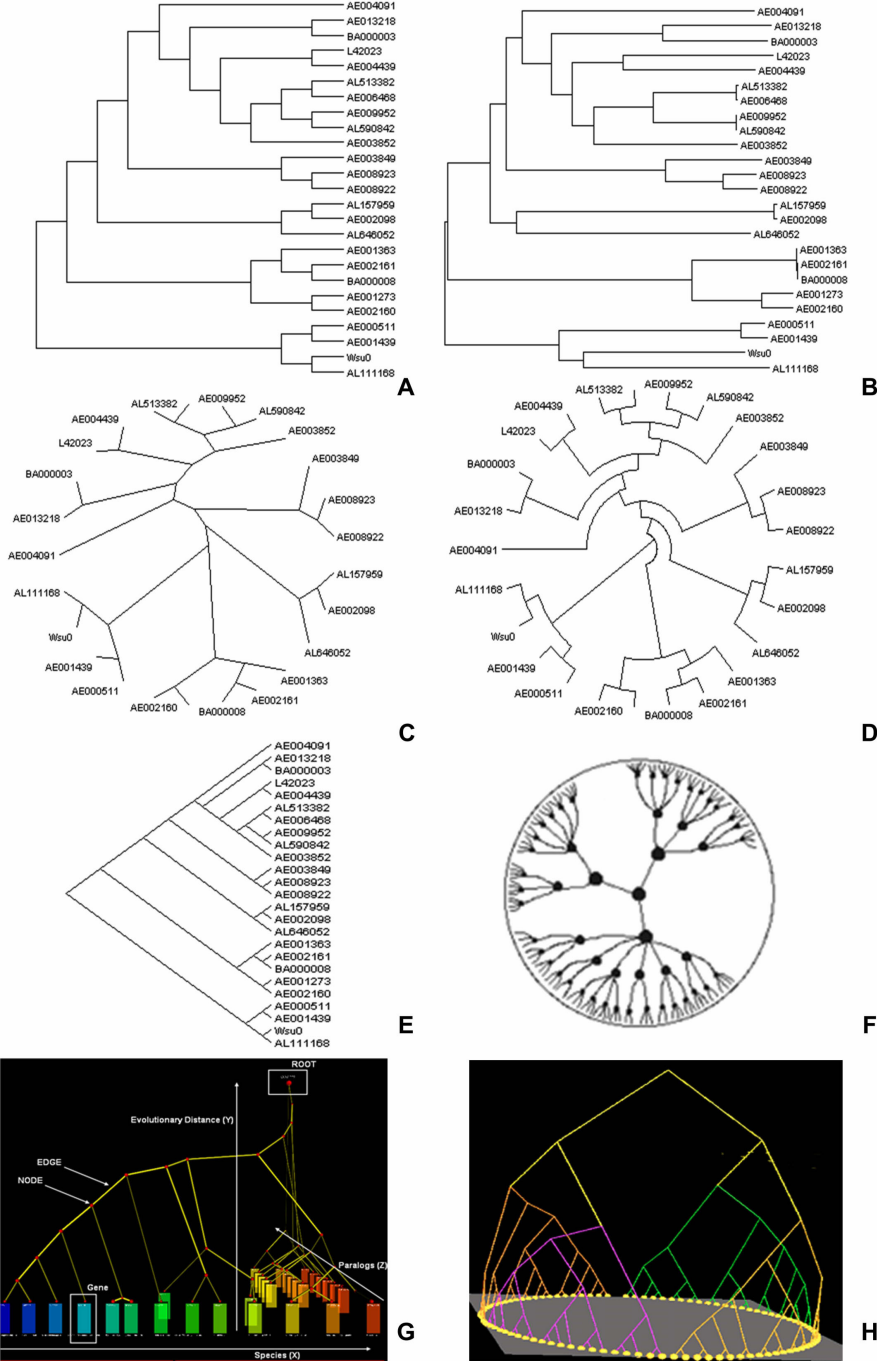
**A) An example of a cladogram representation: a branching diagram assumed to be an estimate of a phylogeny**. B) An example of a phylogram. A phylogram is different from a cladogram with respect to the fact that the branch lengths are proportional to the amount of inferred evolutionary change. C) An example of an unrooted cladogram. An unrooted tree can be rooted on any of its branches, and so there are many rooted trees that can be derived from a single unrooted tree. D) An example of a circular cladogram. These kinds of layout types place the nodes in concentric rings around the center. E) An example of a slanted cladogram. The sloped version of the rectangular layout remains equally informative and efficient. F) An example of a hyperbolic tree. G) 3D Trees by 3DPE (3D Phylogeny Explorer) tool. H) 3D tree visualized by Arena3D [67] visualization tool.

Currently there is a wide variety of tree visualization tools that represent data mostly in 2D dimensions. The vast amount of data makes it necessary that many of these visualizations incorporate efficient layout algorithms that can make navigation easier and the representation of a tree more informative. Figure 1 illustrates different ways for representing relationships between sequences of different organisms.

Each tree can be represented as *cladogram *or *phylogram*. In the first case, a cladogram represents a branching diagram assumed to be an estimate of a phylogeny whereas a phylogram is usually distinguished from a cladogram in that the branch lengths are proportional to the amount of the inferred evolutionary change. Furthermore, each of these types of trees can furthermore be rooted or unrooted. A cladogram or phylogram with a common hypothetical ancestor that equates to the root, which is the node at the base of the tree, is called rooted. A cladogram or phylogram the root of which has not been hypothesized, and for which thus the directions of evolutionary changes among the character-states are not specified, is called unrooted tree. Some of the best known layout algorithms to visualize trees in space and make the graph more informative are the *rectangular phylogram *and *rectangular cladogram *where nodes are aligned in x or y axis the one on top of the other and then the tree is drawn in such a way that it reveals information about the hierarchy. It is not efficient though since it handles the tree as raw data which makes navigation more difficult in cases where the tree consists of thousands of leaves.

*Circular phylograms *and circular cladograms give more intuitive layouts since they use space more efficiently to visualize larger amounts of data. These circular or ring layouts start with the root in the center. The children of the root are placed in one of the concentric rings around the center. The space allocated to each child is proportional to the number of its children. The children that allocate the most space are placed in the outer-most ring.

*Radial *representations use a visual circle to project unrooted trees. This layout is similar to the circular layout but one major difference is that branches can be expanded and nodes can be placed in such a way that clusters or neighbors can be easier visualized. The *radial tree *starts with the root in the center. The children of the root are placed in the inner-most ring. The angle occupied by a child is proportional to the space required by the node.

An ever more efficient layout to visualize data is to use a *hyperbolic space *so the nodes can be enlarged or minimized according to their coordinates. A user can in this way navigate and place the nodes in such a position that the neighborhood of interest is highlighted and enlarged.

In case of larger data sets 3D space and *treemaps *are also used. *Treemaps *display hierarchical trees as a set of nested rectangles or circles [6]. Each branch of the tree is represented by a rectangle or a circle and is then tiled with smaller rectangles or circles representing sub-branches. Branches and sub-branches often follow different color schemes and the area that each leaf rectangle covers is proportional to its dimension. Treemaps can be easily extended for 3D visualization. They are very suitable for pattern recognition by humans and they use space very efficiently so that thousands of data can be visualized simultaneously. The best known algorithms for tiling rectangles efficiently are BinaryTree, Ordered, Squarified and Strip. Treemaps were initially developed by Shneiderman and Johnson [7,8].

Over the last few years, Graphical Processing Unit (GPU) power has increased, therefore 3D graphic programming has become more feasible in terms of memory allocation, calculations and processing speed. 3D space can definitely host larger amount of data but in the case of tree visualization, it is not always well accepted by the community. In later sections, such tools that are able to visualize trees or hierarchies in 3D space are indicated as well.

## Standard tree file formats

In this section we present the available text computer readable file formats that are used to save and load trees. As discussed below, a large variety of tree-viewers exist, which come with distinct and complementary strengths and functionalities. In practice, the only way to integrate features of different tools, including handling of rooted as well as unrooted trees, drawing clusters using different thresholds, changing branch order, expanding and collapsing of trees in various nodes and re-rooting, is based on transforming the tree description across some common file formats. Compared to the format diversity associated with network visualization, the file format landscape around tree viewers is fairly uniform. While a variety of computer readable formats exist, most phylogenetic trees are described using either the New Hampshire/Newick [9], the NHX extended Newick file format or the Nexus [10] file format. In addition, available converters that are able to transform information from the one file format to the other are mentioned in this section, as well as some parsers that are developed to read and save these file formats.

### New Hampshire format

**Figure 2:**
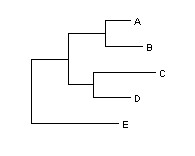
A simple tree example described in Newick format: (((A:0.2, B:0.3):0.3,(C:0.5, D:0.3):0.2):0.3, E:0.7):1.0;

The New Hampshire format, also referred to as Newick, relies on strings of text in order to encode tree representations (see figure 2). However, this format does not impose a uniquely defined representation for a given single tree topology, as the same biological information can be saved and loaded in the shape of various text strings and tree representations. Thus, each tree can be represented by more than one Newick formats. One of the reasons for that is that the left-right order for the positioning of the descendants of a node affects the representation, even though it is biologically not interesting. In addition, users may want an unrooted tree representation, in which case the simple convention is to arbitrarily root the tree. The Newick format description relies on the use of commas and parentheses to define the pairs of nodes to be displayed as connected: a pair of comma separated nodes is enclosed within matched parentheses to indicate that these nodes have a common ancestor. The length of a branch can be incorporated next to a node name followed by a colon.

### The NHX format

In spite of the fact that it follows the same encoding principles as the original Newick format, the New Hampshire eXtended - NHX - format is richer and can incorporate additional information about tree nodes and branches. The most important extension of NHX, as compared to Newick, is the introduction of tags and meta-data support in order to associate various data fields with a node of a phylogenetic tree. NHX format is universal and it is suitable for describing annotated phylogenies. In comparison with the simpler New Hampshire format, in NHX the order of tags can be flexible, the length of string based data is unlimited (such as species and descriptions), both internal and external (root and leaf) nodes can be tagged, a bigger variety of tags is offered for different data fields and there can be an arbitrary number of children per node.

### The Nexus format

The Nexus file format is very similar to the Newick format and it was designed to support meta-data for each incorporated data type. Examples would be the support of alignment sections allowing users to import sequence alignments, the support of translate sections allowing the incorporation of additional information about each individual leaf, or the support of tree sections giving the possibility to provide the hierarchical structure of the tree in text. It was initially introduced by PAUP -- Phylogenetic Analysis Using Parsimony [11] which is a widely used software package for the inference of evolutionary trees and MrBayes [12] which is a program for the Bayesian estimation of phylogenies.

Several tools including MrBayes [12], PAUP [11], PHYLIP [13], ITOL [14], PROTML, TREE-PUZZLE [15] recognize both the Newick and NHX format. It is the only tree file format readable by the PHYLIP programs drawgram, drawtree, and retree and can be imported and exported by almost every other program. The NEXUS [10] format incorporates the Newick [16] text string along with other commands required by other programs such as PAUP* [11]. Most tree viewing programs can handle NEXUS [10], one exception being the PHYLIP package [13]. There are many tools that convert NEXUS into NHX files, and vice versa (one can be found under http://www.ii.uib.no/~matthewb/tools/tree_convert_in.cgi). The easiest way to switch tree file formats is to use the already existing modules offered by the open source projects BioPerl [17] or BioJava [18]. Users will also have the capability to save trees in XML like formats like SVG or other forms like PDF, JPG or PNG. Treeplot is another good conversion tool to convert PHYLIP [13] format to Postscript (.ps), Adobe Illustrator .ai , Computer Graphic Metafile .cgm, Hewlet Packard Graphic Language .hpgl, xfig file .fig , image file .gif and PBM Portable aNy Map file .pnm .

## Trees in Life Sciences

In this section, examples of different life science areas for which the tree representation is of importance are presented. We shortly describe the performed analysis type and show how the tree visualization can be the appropriate or standard way to display, describe and process the respective data.

### Phylogenetic analysis - evolution studies

Phylogenetic analysis aims to study the evolutionary relationships among the different organisms. It is the study of evolutionary relatedness among various groups of organisms (for example, species, and populations). Owing to the technological advancement of the sequencing techniques in molecular biology and the ability to collect large amounts of data (DNA or amino acid sequences) from disparate organisms, phylogenetic analysis and evolutionary studies are still of highest interest. Currently, similarities among species or populations are being primarily calculated with the use of molecular sequencing data or morphological data matrices, whereas in the past they were based on anatomical features. Due to the fact that evolution takes place over thousands of years, the changes are not obvious and cannot be observed immediately. Therefore scientists must reconstruct phylogenies by inferring the evolutionary relationships among organisms that exist nowadays. Fossils can aid with the reconstruction of phylogenies; however, fossil records are often too poor to be of good help. This means that evolutionary analysis is restricted to analyse present-day organisms to identify their evolutionary relationships.

Inevitably, the nature of phylogenetic studies and the above mentioned aspects, impose the tree-like representation, the so called 'phylogenetic tree', as the most informative and thus popular graphical way to describe the discovered relationships and similarities. Since evolution is regarded as a branching process, whereby populations are altered over time and may diverge into separate branches, hybridize together, or terminate by extinction, the tree visualization is the best representation to describe this procedure. In a phylogenetic tree, every leaf node represents a species, each edge denotes a relationship between two neighboring species and the length of an edge indicates the evolutionary distance among them. Evolutionary studies aim to calculate and present an accurate tree of life, in which all existing species can be incorporated. The most commonly-used methods to infer phylogenies and compare or cluster species include maximum parsimony, maximum likelihood, Neighbor Joining [19], UPGMA [20] and Monte Carlo or MCMC-based Bayesian inference techniques [21]; see table 1.

**Table 1:** Short description of the most commonly used methods to infer phylogenies.

Method	Input	Description
Neigbor-Joining (NJ)	Distance Matrix	Iterative clustering method based on the minimum-evolution criterion; the topology with the least total branch length is preferred at each step.
UPGMA		Agglomerative hierarchical clustering based on the average linkage method.
Maximum Parsimony (MP)	Phylogenetic Feature Matrix	Alternative evolutionary trees are generated; the one that satisfies the parsimony optimal criterion is considered as the best estimation: under maximum parsimony, the preferred phylogenetic tree is the tree that requires the smallest number of evolutionary changes.
Maximum Likelihood (ML)		Alternative evolutionary trees are generated; the probability of an evolutionary event at any given point on a tree is stochastically modelled: under maximum likelihood, the preferred phylogenetic tree is the one with the highest likelihood.
Markov Chain Monte Carlo (MCMC)	Both	Bayesian inference method; alternative evolutionary trees are generated combining a posterior distribution for a feature and a model of evolution, based on the prior for that feature and the likelihood of the data, generated by a multiple alignment: unlike MP and ML a set of equally optimal trees may be produced. MCMC simulation is used to sample trees towards a credible subset.

The distance matrix methods are faster, computationally less expensive and can thus be applied in larger data sets. Nevertheless, the other methods are considered to produce better results.

### Proteomics, Transcriptomics and Genomics

In mathematics, a *distance matrix *is a 2D matrix - array that contains the pairwise distances of a set of points. It is therefore a symmetric *N *× *N *matrix, where N is the number of points, containing non-negative values as elements. The number of pairs of points *N *× *(N-1)/2 *is the number of independent elements in the distance matrix. The distance matrix is often used as a synonym for a dissimilarity matrix. The term distance does not necessarily mean distance in Euclidean space. We often use the "distance" as a subjective measure of dissimilarity.

The *similarity *matrix is the opposite concept to the distance matrix. The elements of a similarity matrix contain pairwise similarity measurements of objects. The greater the similarity of two objects is, the greater the value of the measure is. For example, the correlation matrix often may be considered as a similarity matrix of variables - because it is natural to consider pairs of variables with high correlation coefficient values to be more similar to each other than pairs with lower correlation coefficient values.

A *correlation matrix *describes correlations among N variables. It is a symmetrical squared NxN matrix with the (ij)th element equal to the correlation coefficient between the (i)th and the (j)th variable. The diagonal elements are always equal to 1.00 because they are 100% correlated with each other. Many methods of multivariate statistical analysis rely on a correlation matrix as the initial data, e.g. principal component analysis, factor analysis or path analysis.

The *correlation coefficient *indicates the degree of linear relationship between two variables. The correlation coefficient can take values between -1 and +1. The -1 value or else the -100% indicates a perfect linear negative relationship between two variables meaning that these two variables are anti-correlated. The +1 value otherwise +100% indicates the perfect positive linear relationship between the variables. The 0 indicates lack of any linear relationship. A very common measurement to calculate the correlation between two variables is the Pearson Correlation Coefficient.

In bioinformatics, distance matrices are used to represent protein structures in a coordinate-independent manner, as well as the pairwise distances between two sequences in sequence space. They are used for structural and sequence alignments, and in a vast variety of methods used in proteomics, transcriptomics and genomics Chip-Chip, mass spectrometry and microarray technologies. To analyze these data, usually a distance or a similarity matrix is created and often followed by some grouping or clustering methodology. A typical example for these distance matrices can be produced by applying sequence alignment methods, like a Smith-Waterman algorithm to a set of related DNA or protein sequences [22]. The resulting alignment scores represent how similar two sequences are and thus are used as measures to construct the matrix. Another example could be from microarray studies, where experimentalists calculate the expression profiles of genes at distinct time steps. For various time points they determine the expression of each gene. By collecting these values within time and by using the statistical Pearson Correlation Coefficient (PCC) method, one can detect how much related two genes are according to their expression behavior. The PCC measurement can, as in the sequence alignment case, be considered as the similarity value basis to construct the matrix upon.

The common visualizations used for the display of results and analysis outcomes are either trees or the, so called, heat maps. A *heatmap *is a graphical two dimensional representation of data where the values of a variable are represented as colors; the coloring scheme may be combined with alteration in their intensities in order to display the corresponding distribution of the values of the respective data, parameters or measurements of interest. Heatmaps themselves, as well as analysis on top of them, most frequently are appropriate for clustering and tree visualization. For example, hierarchical and k-means clustering are widely used techniques in microarray analysis whereas UPGMA [5,6] has been mainly developed for processing proteomic data (e.g. mass-spectrometry data). Commonly the analysis performed on heatmaps includes clustering of the visualized data per measured dimension or via the previously mentioned treemaps.

### Sequence alignment

There are many algorithms and tools available for the basic problem of aligning multiple sequences, such as ClustalW [23], MUSCLE [24,25], BLAST [26], and the T-Coffee suite [27]. The results produced by these tools are particularly useful to a wide variety of life sciences fields, from phylogenetic analysis to motif identification. Alignment can be performed between two or more (all against all) protein or DNA sequences. For example, Needleman-Wunsch [28] or Smith Waterman [22] are dynamic programming methodologies that provide scores to show the degree of similarity between sequences; an overview regarding sequence similarity methods can be found in [29-31].

Clustering and tree structures are used as part of the alignment algorithms, as well as for the processing and representation of the alignment results. Commonly, the sequence alignment scores and similarity measures [32,33] are being considered to be the basis for the creation of the corresponding distance or similarity matrices upon which then a clustering algorithm is applied. Such sequence alignment analysis results are commonly visualized using tree representations.

### Clustering Analysis

As mentioned previously, clustering methodologies can be applied to a wide range of biological study cases, such as microarray analysis, sequence analysis and phylogenetic analysis. Some of the most common clustering algorithms used in biology are UPGMA [20,34], Neighbor Joining [19,35] and Hierarchical Clustering [36,37], all of which represent their clusters as tree structures. The input to these algorithms is usually a similarity or a distance matrix. Hierarchical Clustering [36,37] begins with the individual elements and progressively merges clusters according to the defined distance criterion. Depending on the distance criterion used for populating the clusters, hierarchical clustering is divided into the following major categories and their alterations: single-linkage clustering, complete-linkage clustering and average-linkage clustering; in Table 2 some of the most common agglomeration methods are described. UPGMA [20,34], which stands for Unweighted Pair Group Method with Arithmetic mean, in phylogenetic analysis assumes a constant rate of evolution and is not a well-regarded method for inferring phylogenetic trees unless this assumption has been tested and justified for the data set being used. Neighbor Joining [19,35], on the other hand, although it constructs the tree in a step-wise fashion by utilizing the input distance matrix and by joining the closest neighbors, is more popular in phylogenetic analysis as it can be used on very large data sets for which other methods (e.g. minimum evolution, maximum parsimony, maximum likelihood) are computationally prohibitive. Last, while the Neighbor Joining and Hierarchical Clustering methods are widely used to group microarray data, UPGMA is preferable to cluster proteomics data, for example produced by mass spectrometry.

**Table 2:** Description of common agglomerative metrics used as cluster merging criteria

		Agglomerative Methods
Abbreviation	Full Name	Description
single	Single Linkage	Merge those clusters for which the minimum distance between their elements is the least one.
complete	Complete Linkage	Merge those clusters for which the maximum distance between their elements is the least one.
average	Average Linkage	Merge those clusters for which the mean distance between their elements is the least one.
centroid	Centroid Method	Merge those clusters for which the (squared) Euclidean distance between their centroids or means is the least one.
median	Median Method	Merge those clusters for which the Euclidean distance between their weighted centroids is the least one; called median because the centre of each new cluster is based on the combination of the centroids of the merged groups.
ward	Ward's Criterion, or else Ward's Minimum-Variance Method	Merge those clusters for which the increase in variance for the resulting group is the least one.
EML	EML	Merge those clusters that maximize the likelihood at each level of the resulted hierarchy; similar to Ward's method but removes the bias toward equal-sized clusters.
density	Density Linkage	Merge those clusters for which the probability density estimate for the resulting group is maximized; consists of two steps: 1. the dissimilarity measure is based on reciprocals of the estimates of the density midway between the members of each cluster within a defined area or otherwise is infinite, 2. a single linkage cluster analysis follows. (Examples of different types of density methods are the kth-nearest-neighbor, the uniform kernel and the Wong's hybrid ones which among others differ with respect to the neighbourhood within which the density is measured)
mcquitty	McQuitty's Similarity Analysis	Merge those clusters for which the average of their distances, or else the distance of the resulting group, from the remaining ones is minimal.

### Ontologies - Hierarchies

An ontology is a formal representation of a set of concepts within a domain and the relationships between those concepts. In life sciences, there is a strong trend towards the creation of unified and controlled vocabularies (e.g. Mesh, UMLS [2], OBO-Open Biomedical Ontology, part of which is GO-Gene Ontology, etc.). These ontologies are very big and are organized into ordered hierarchies. Although these ontologies are in some cases not organized as trees, the tree is the most commonly used representation for their visualization as it can preserve the hierarchical order of the relationships among the concepts displayed; usually only a subset is manipulated which in many cases results in a real tree-graph.

## A survey of tree visualization tools

The purpose of the preceding short overview has been to understand how and why the study and visualization of trees in the biological context is crucial. The next section gives an overview of the most widely used tree-viewers for the analysis and visualization of data, primarily developed for phylogenetic analysis purposes, and covers mostly recent visualization tools, i.e mainly developed in this decade. The purpose is not to compare, but rather to assist in selecting the appropriate tool for a study.

### Dendroscope [38]

It is a java platform which is able to visualize rooted phylogenetic trees in 2D very efficiently. It is recommended for very big datasets with hundreds of thousands of taxa. With Dendroscope the user can edit and manipulate the tree interactively. For example, it supports functionalities like zooming in/out, subtree collapsing and expanding, subtree coloring or re-rooting of the tree. Furthermore, one can query the data using regular expressions. Last, it supports several layout algorithms such as the rectangular, slanted, circular and radial views. It is a tool that can be of general use since it accepts all of the aforementioned Newick, Nexus and NHX formats. Nevertheless, it is not connected to any data sources and it does not offer clustering or statistical analysis capabilities.

### iTOL [14]

It is a non-open-source web application to display and manipulate phylogenetic trees in 2D. ITOL uses Shockwave Flash and Javascript to display trees and it reads the Newick and the Nexus file formats. It comes with both rectangular and circular-ring layouts and supports subtree coloring, tree re-rooting, branch pruning, expansion and deletion of subtrees and expansion and deletion of leaf nodes. It is interactive and allows flexible exploration, annotation and visualization of statistics describing phylogenetic trees. It is mainly designed for mid-size datasets, up to approximately few thousands of taxa, and is not recommended for very big datasets. A prime application of iTOL visualization is the latest version of the Tree of Life, which shows the evolutionary relationship between 191 different species [39].

### HyperTree [40]

It is a freely available java application which was mainly developed for viewing, editing and manipulating big data sets in 2D. It is reads and writes trees in PHYLIP file format. The particular strength of HyperTree is the implemented efficient scaling algorithms for large phylogenetic trees. It uses a hyperbolic view in order to facilitate understandable views even for magnitudes of data for which visualization becomes difficult. It is interactive: mouse controls allow zooming in/out, rotation and dragging of the tree, subtree coloring, branch labeling, search and selection of a set of nodes and cluster generation results compatible to other applications. It currently does not support any other layout besides that of the hyperbolic space and it does not allow node editing or annotation.

### NJPlot [41]

It is an open source standalone application that was mainly developed for rooted and unrooted tree visualization in 2D. It reads the Newick format as input and it allows zooming, branch swapping, display of bootstrap scores and it supports drawing of multi-branched trees with or without branch lengths. It comes with limited functionality comparing to other tools and is not recommended for large datasets. Currently it does not support layout algorithms and nodes cannot be annotated or edited. Nevertheless, it is applicable to rooting unrooted trees obtained from the various tree-building methods.

### HyperGeny [42]

HYPERbolic phyloGENY viewer is a platform independent java application that was mainly developed for hyperbolic visualization of large tree structures in 2D, available for academic use only. It receives as input and exports trees and subtrees in the Newick format only. It supports functionalities like zooming in/out, subtree collapsing and expanding, subtree coloring, bootstrap and label value visualization or label selection. Nodes cannot be edited or annotated and currently it does not come with statistical or phylogenetic analysis methods. It comes with its own API to be easily integrated into other projects.

### CTree [43]

It is java application mainly developed for viewing, analyzing and editing phylogenetic trees in 2D. It can read and write trees in Newick and .pdf format, respectively. It is not very interactive but is able to handle large data sets. It supports, tree re-rooting, multiple tree loading, swapping the order of sibling strains, tree and subtree coloring, visualization of labels, bootstrap values, evolutionary distances and searching capabilities for node selection. Its main strength is its ability to recognize and color clusters on the tree using heuristic algorithms. It also supports coloring and display of manually defined clusters. It is one of the few tools that come with statistical analysis of the tree, such as calculation of subtype diversity ratio and subtype diversity variance distributions. It does not come with many layout algorithms and from that perspective it is not recommended for large datasets although it is suitable for clustering detection and visualization.

### Phylowidget [44]

It is an open source java standalone and web application mainly developed for viewing, editing, and publishing 2D phylogenetic trees online. It recognizes trees in Newick, NHX and Nexus format and can export high resolution images ready for publishing. It is interactive supporting the functionalities that most other visualization tools offer and can be recommended for big datasets since it utilizes many layout algorithms, like Dendroscope does. Its main strength is that it can produce high-quality views for publishing and it can be easily integrated to other projects through a URL-based API. Another, similar to Phylowidget, tool that can also produce high quality and ready for publication images is TreeGraph [45].

### BAOBAB [46]

It is an open source java and it can read trees in Newick, Nexus and XML format It allows the creation and the interactive modification of a tree, the addition and deletion of nodes, moving of branches and nodes, changing of leaf names and of parameterization settings. Baobab allows users to manage sequence files along with a tree. Supported formats for input and output include Fasta, Phylip and Clustal [23]. It does not come with many layout algorithms and it is not recommended for large scale data visualization. It is a suitable tool, though, for editing and annotating phylogenetic trees.

### TreeIllustrator [47]

It is a freely accessible java application mainly developed for visualization and customization of 2D phylogenetic trees. It can take as input trees in Newick format and it comes with a variety of functionalities like the dragging of nodes, different tree shapes, zooming and searching capabilities, and support for large trees. It is rich in layout algorithms and it contains a simple and effective method that compares a custom tree with the Tree of Life, by detecting incongruence.

Other widely used tree visualization tools are mentioned below; see Table 3 and 4. The following tree-viewers come with distinct advantages and short-comings that make them suitable for different kinds of problems, data types and quantities. Many of them serve different purposes and they can be applied differently according to the users' interests. Besides the aforementioned tools, widely used software for phylogenetic pipeline analysis is the BAOBAB [46] for statisctical analysis, BioNumerics [48] which offers integrative analysis with other bioinformatics tools, COMPONENT [49], PAL [50], POWER [51], MEGA [52-55], Mesquite [56] for evolutionary analysis, Geneious for phylogenetic sequence analysis, MacClade [57] and TreeQ-Vista [58], PAUP [11] for molecular, morphological and/or behavioral data analysis to infer phylogenetic relationships.

**Table 3:** Summary of representative tree visualization tools and categories, as they are described inthe main text of the article.

	Popular Phylogenetic Tree Viewers Applicable to Generic Use				
Tool	Availability	File Format(s)	Export	Data Set Size	Layouts
Dendroscope	Free for Academic Use	Newick, Nexus and NHX	.eps, .svg, .png, .jpg, .gif, .bmp, .pdf.	large	rectangular, slanted, circular and radial
iTOL	Online version Free	Newick and Nexus	.svg, .png, .eps, or .pdf	mid-size	rectangular and circular-ring
HyperTree	Free for non-commercial Use	PHYLIP file format	PHYLIP file format	large	hyperbolic view
NJPlot	Free	Newick	.pdf and .eps	small	rectangular
HyperGeny	Free for Academic Use	Newick	Newick	large	hyperbolic view
CTree	Free	Newick	.pdf	large	rectangular and unrooted
Phylowidget	Free	Newick, NHX and Nexus	.jpeg, .pdf and .png	large	rectangular, diagonal, circular and unrooted
BAOBAB	Free	Newick, Nexus and XML	Newick, XML and Pag	large	rectangular and unrooted
TreeIllustrator	Free	Newick	.eps, .jpeg	large	rectangular, radial and slanted cladograms

The table is organized neither with purpose to compare nor in manner adequate for the scope of an absolute evaluation. All tools display different interactivity and tree manipulation functionalities making each one suitable to alternative case studies. In addition, generic use, i.e. not only phylogenetic related, may apply, an issue that depends on the content of the data incorporated by a user in the acceptable per tool input file formats and tree management possibilities.

**Table 4:** This table presents a list of widely used visualization tools that most of them are not presented in the text.

Other categorizations	
**3D Visualization**	Paloverde [68], Walrus[78], Arena3D [67], Arbor3D and 3DPE
**Hyperbolic Space**	H3Viewer [69], HYPERTREE [40], Walrus [70], Hypergeny [42]
**Cluster Visualization**	TreeTracker [71], TreeMe [72], CTree [43] and 3DPE [73].
**Tree Comparison**	COMPONENT [49] and Phylo-comparison [74].
**Tree Editing**	TaxonTree [75], TreeEdit [76], TreeDyn [77], BAOBAB [46], MacClade [57], Mavric [78], TreeGraph [45]
**Tree Annotation**	TaxonTree [75], TreeEdit [76], TreeDyn [77], BAOBAB [46], MacClade [57], Mavric [78], TreeGraph [45], Ape [79], iTOL [14], Mesquite [56], PAL [50], PhyloGena [80], PoInTree [81], THEA [82], TreeDyn [77], Treemap [83] and TreeQ-Vista [58].

The tools are organized according to their properties and the functionality that they offer.

### A survey of tree related biological analysis and manipulation tools

In the following section, software, tools and packages or libraries that offer the ability to perform various types of tree related analysis for biological data are presented; see Table 5 and 6; not all available tools are listed but we tried to include the ones which are widely used and accepted by the scientific community. Many of the tools have their strength in the analysis of the data and are not as interactive or user-friendly in the visualization as the aforementioned tools in the previous section. This gap between analysis and tree visualization is stressed and should be resolved. Thus, purpose of the current section is to point out how life sciences users can currently bridge the tree associated chasm to both analyze and visualize their data.

**Table 5:** Summary of representative software related to analysis applicable to tree visualization.

Software	Availability	Visualization	Interfaces	Tree Format	Clustering Analysis	Phylogenetic/Evolutionary Analysis	Microarray Analysis	Sequence Alignment Analysis
**MATLAB**	Commercial	Yes	BioPerl, BioJava, C, Java, Perl	Newick	Yes	Yes	Yes	Yes
**MEGA**	Free for use in research	Yes	PAUP, PHYLIP	Newick, Nexus	Yes*	Yes	No	Yes
**PHYLIP**	Free	Limited	written in C	Newick	Yes*	Yes	No	No
**Geneious**	Commercial; Free trial available	Yes	PAUP*, MrBayes, PHYLIP, MEGA	Newick, Nexus	Yes*	Yes	No	Yes
PAUP*	From version 4.0 on must be purchased	Limited	PHYLIP, MEGA	Nexus	Yes*	Yes	No	No
**MrBayes**	Free	Limited	written in C	Nexus	No	Yes	No	No
**HCE**	Free for use in research	Yes	Does not apply; Output: tab delimited .txt or .bmp files.	Does not apply; Data Input: tab delimited .txt or .xls files.	Yes	No	Yes	No
**TM4**	Free for use in research	Yes	Does not apply; Output: tab delimited .txt or .tiff or .env, TM4 specific etc files.	Does not apply; Ability to save clusters and trees in Newick, Nexus.	Yes	No	Yes	No

The table is not a complete summary but an overview of the features related and mentioned to the main text as well as their connectivity. Last Yes* for clustering refers to distance matrix based phylogenetic tree construction methods such as NJ and UPGMA.

**Table 6:** It refers to analysis that each package can by itself perform irrespective of whether it can receive as input results produced from another tool for a specific category of analysis.

Bio* and Open-source projects: limited or no visualization	
Project	Comments
**R**	Interfaces all major prog. languages, such as MATLAB, Perl, Python, Java, C, C++ and Fortran; wide bioinformatics, statistical and clustering analysis possibilities.
**Bioconductor**	Relies on R; appropriate towards statistical analysis of microarray experiments.
**BioPerl**	Relies on Perl; phylogenetic analysis, multiple sequence alignment and microarray analysis are facilitated; well-established interfaces to other prog. languages.
**BioJava**	Relies on java; mainly sequence analysis; low compatibility with other languages and limited functionalities compared to BioPerl.
**BioPython, BioRuby, BioBike, BioLisp**	Reliance on Python, Ruby, BioLisp, Lisp respectively; apart from the main sequence alignment, among others, microarray analysis is available too.

### Generic Tools: Sequence alignment, Microarray and Phylogenetic analysis

#### 1. Matlab

MATLAB http://www.mathworks.com/ is a high-level mathematical language and interactive environment with which one can perform computational tasks. There is specific computational biology support for the analysis, visualization and simulation of biological data and systems via two toolboxes: the Bioinformatics and the SimBiology toolboxes. Some of their features are: sequence-, phylogenetic-, microarray- and mass-spectrometry- data analysis as well as programming interface to the described later BioPerl [17] and BioJava [18] projects. For the purpose of the current survey, MATLAB provides the ability to visualize data and comes with a multiple sequence alignment viewer, phylogenetic tree tools and methods for graph analysis. In spite of its broad usefulness in providing the ability to develop new algorithms or to share and deploy new applications, the tree visualization part offered is, nevertheless, limited and cannot handle large amounts of data.

With MATLAB one can use various tree-format strings and also manipulate tree data, for example by selecting branches and leaves using a specified criterion or by removing nodes and comparing trees. Furthermore, both phylogenetic and microarray analysis capabilities are available. Specifically, with MATLAB one can use functions for tree building and processing, such as drawing phylograms, cladograms, or radial treeplots and estimate the substitution rates, read and write Newick-formatted tree files, calculate the pairwise distance matrices for given biological sequences or view the tree in a MATLAB-oriented interactive GUI that allows to view, edit, and explore the data, prune branches, reorder, rename, and explore distances. In addition, MATLAB comes with a variety of clustering algorithms and lots of functionality to process microarray data like Affymetrix GeneChip, ImaGene result, SPOT, Agilent microarray scanner, GenePix GPR or GAL files as well as Gene Expression Omnibus (GEO) data. Last, MATLAB is also compatible with other programming languages to communicate with C, Java, Perl and Matlab code. Nevertheless, although the platform of MATLAB is recommended for scientific analysis, it is not freely available.

#### 2. MEGA 4: Molecular Evolutionary Genetics Analysis

MEGA [52-55]http://www.megasoftware.net/ is an integrated tool for conducting automatic and manual sequence alignment, inferring phylogenetic trees, mining web-based databases, estimating rates of molecular evolution, and testing evolutionary hypotheses. To the concern of the current review, MEGA comes with several methods for clustering and tree construction, like Neighbor-Joining [59], Minimum Evolution method [60,61], UPGMA [20], Maximum Parsimony [62], Bootstrap Test of Phylogeny [63], Confidence Probability Test, Consensus tree construction, Condensed tree construction. It also comes with tree explorers, phylogeny display and graphic printing, on-the-spot taxa name editing, multiple phylogeny views, linearized tree, and estimation of divergence time by calibrating molecular clock and so on. MEGA can read and write the Newick format and save trees in files as EMF drawings. Users can adjust and define the branch lengths, add scaling and shifting bars interactively, collapse branches or groups of braches, display subtrees or view multiple trees in different viewers. Furthermore users can edit and modify the trees by flipping, re-rooting, adding marker symbols to names or displaying multi-colored schemes. In addition, one can change the tree size, the vertical separation between taxa and the horizontal size or the tree shape. Users can have multiple tree displays, save tree sessions for future display, display images on tree for groups and taxa. The ability to read sequencer, MEGA, NEXUS, FASTA, and other formats, importing Data from other formats (Clustal/Nexus/etc.) and export to PAUP and PHYLIP is also a strong point of MEGA.

#### 3. PHYLIP

PHYLIP http://evolution.genetics.washington.edu/phylip.html is a free package of programs for inferring phylogenies. Methods that are available in the package include parsimony, distance matrix utilization and likelihood methods, including bootstrapping and consensus trees. Data types that can be handled include molecular sequences, gene frequencies, restriction sites and fragments, distance matrices, and discrete characters. Trees are supported in the Newick format.

#### 4. Geneious

Geneious http://www.geneious.com/ is an integrated, cross-platform bioinformatics research software suite that combines major analysis tools. Geneious is a commercial product and a free trial is available. Features include sequence alignment and phylogenetic analysis. Geneious combines a number of visualization tools for different types of data and analyses. In specific, there is an interactive phylogenetic tree viewer and builder. In addition, there are available plugins for PAUP* and MrBayes.

#### 5. PAUP

PAUP http://paup.csit.fsu.edu/ is a software package for inference of evolutionary trees. Pairwise distances can be summarized in a table or be used to construct UPGMA and neighbor joining trees. Furthermore, PAUP* provides a wide range of pairwise tree-distance measures, from simple absolute differences to more complicated model-based corrected ones. In addition, PAUP* can use the minimum evolution and least-squares functions to evaluate trees under a distance criterion. Input files of PAUP* are in the NEXUS file format and can contain data (the sequences or the distance matrix) or commands. PAUP analyzes data using different optimality criteria (parsimony, likelihood, and distance) and search methods (exact and heuristic) for creating the optimal trees. The created trees can be viewed in different levels of resolution and description. PAUP* can save trees in different formats: PICT (Mac only), NEXUS, Freqpars, Phylip, and Hennig86. In addition, PAUP proposes third part tree viewers for higher resolution, like TreeView.

#### 6. MrBayes: Bayesian Inference of Phylogeny

MrBayes [12]http://mrbayes.csit.fsu.edu/ is a program for the Bayesian estimation of phylogeny. The program takes as input a character matrix in a NEXUS file format, for example with DNA sequences, and among the output it also generates the Nexus formatted phylogram that corresponds to the user specified parameterization. The trees like in PAUP* will also be printed to a file that can be read by tree drawing programs such as TreeView, MacClade, and Mesquite.

#### 7. Hierarchical Clustering Explorer (HCE)

HCE [64,65]http://www.cs.umd.edu/hcil/hce/ stands for Hierarchical Clustering Explorer for Interactive Exploration of Multidimensional Data, such as microarray experiment data sets. HCE applies clustering without a predetermined number of groups, and then enables users to determine themselves the acceptable limits via interactive visual feedback, like dendrograms and colorful mosaics. In summary, with HCE one can display hierarchical clustering results and dendrograms or color mosaic displays for multidimensional data sets. An interactive visualization allows users to control the distribution and ranking over one or both dimensions altering thus the clusters. Statistical feedback helps the user to conclude. For the tree visualization part there is a minimum similarity criterion that the user can change and correspondingly view the new formed clusters. Different coloring of the subtrees makes cluster visualization easy. HCE is free for academic and/or research purposes.

#### 8. Microarray sotware suite

TM4 [66]http://www.tm4.org is an open-source, free software. The TM4 suite of tools consists of the following four major applications: Microarray Data Manager (MADAM), TIGR_Spotfinder, Microarray Data Analysis System (MIDAS), Multiexperiment Viewer (MeV). There is also a Minimal Information about a Microarray Experiment (MIAME) compliant MySQL database. All applications are freely available to the scientific research community. TM4 incorporates algorithms for clustering, visualization, classification, statistical analysis and biological theme discovery. TM4 has its own file format, the mev file format. There is a converter that transforms into the mev format data from Genepix, ImaGene, ScanArray, ArrayVersion and Agilent files. Affymetrix data files can be loaded directly into TIGR MeV.

### Bio* and Open-source projects

#### 1. The R Project for Statistical Computing

R http://www.r-project.org/ is a free software environment for statistical computing and graphics. It compiles and runs on a wide variety of UNIX platforms, Windows and MacOS. There are R interfaces for all major programming languages, such as MATLAB, Perl, Python, Java, C, C++ and Fortran. The R package system itself provides implementations for a broad range of statistical and graphical techniques, including modeling and cluster analysis. R is popular in the bioinformatics world as it is free and open source (in contrast to for example, MATLAB). It is highly recommended for biological analysis since lots of documentation is available online.

#### 2. Bioconductor http://www.bioconductor.org/

Bioconductor http://www.bioconductor.org/ is based on the R project. Bioconductor is an open source and open development software project that aims to provide access to a range of statistical and graphical methods and tools for the analysis and comprehension of genomic data. For example, there are analysis packages and statistical or graphical methods available for: preprocessing Affymetrix and cDNA array data; identifying differentially expressed genes; graph theoretical analyses; plotting genomic data. Bioconductor is appropriate towards statistical analysis of microarray experiments, array preprocessing and quality control, within- and between-array normalization, binding of covariate and design data to expression data, and downstream inference on biological and clinical questions.

#### 3. BioPerl

BioPerl [17]http://BioPerl.org is a toolkit of bioinformatics Perl programming language modules. Among others, there are modules related to phylogenetic analysis, multiple sequence alignment and microarray analysis. BioPerl is primarily appropriate for processing sequence data and interfacing to sequence databases, with support for sequence visualization and queries for external annotation. For example, one can generate a phylogenetic tree from protein sequence alignment data using parsimony criteria, generate a pairwise sequence distance matrix based on an alignment of protein sequences, create a phylogenetic tree from the output of the calculated distances using either the Neighbor-Joining or UPGMA methods or calculate a consensus tree typically for a set of bootstrapped replicates; the distance matrix produced is PHYLIP friendly. Bioperl provides reusable Perl modules facilitating sequence manipulation, accessing of databases using a range of data formats, execution and parsing of the results of various molecular biology programs including Blast, ClustalW, TCoffee, Genscan, ESTscan and HMMER, PHYLIP, BLAST, GENSCAN, and the EMBOSS suite. Bioperl is highly portable, open and free with well-established interfaces to other programming languages.

#### 4. BioJava

BioJava http://BioJava.org is an open-source project dedicated to providing a Java framework for processing biological data. Features include objects for manipulating biological sequences, file parsers and tools for making sequence analysis GUIs. BioJava is licensed under LGPL 2.1 and runs on any computer with a Java virtual machine complying to the Java 2 Standard Edition (J2SE) 1.4 (or later) specifications. Java implementations for Linux, Windows, and Solaris are available and recently for MacOS X. There are efforts for improving Biojava compatibility with other languages. However, the BioJava Framework has currently limited functionalities as compared to the BioPerl toolkit. It is recommended for Java programmers but it is limited comparing to BioPerl module.

#### 5. BioPython - Bioruby - BioBike - BioLisp

The Biopython Project http://BioPython.org is an international association of developers of freely available Python tools and libraries for computational molecular biology. Biopython functionality includes interface to Clustalw alignment program and code to perform classification of data. The GUI is restricted to basic sequence manipulations, translations, BLASTing, etc. BioRuby project http://bioruby.org/ provides an integrated environment in bioinformatics for the Ruby language (E.g. Sequence analysis, BLAST, HMMER). A graphics library is available but not specific to trees. The entire BioRuby package is written in pure Ruby, so there are not any OS dependent issues. The BioBike system http://nostoc.stanford.edu/Docs/ is a biology-specific programming language. BioBike has built-in all of the typical bioinformatics tools (Blast, Clustal, etc.). The main BioBike language is called BioLisp - a dialect of common lisp with biological functionality added in. BioBike is the environment in which one can write and execute BioLisp code. With Biolisp, among others, one can perform simple biological natural language processing on PubMed, work on sequences, represent and search graphs, produce trees and perform microarray data clustering. To our knowledge, No visualization is provided, however.

## Discussion

The data production rate in modern molecular biology is scaling up dramatically. The increasing use of high-throughput technologies multiplies the amount of data generated and rapidly fills the databases. The need to sequence more species and create bigger and more precise trees of life, which will contain as many species as possible, in order to reveal biological information about the evolutionary origin of human and other organisms is becoming more and more challenging. Cost, efficiency and scale of biological experiments can only improve and produce larger amounts of results that make the visualization of data a major bottleneck in systems biology and other large-scale approaches. The amount of data and their heterogeneity pose a great challenge and therefore the development of efficient visualization tools that can construct representations of data on-the-fly has become a critical objective for bioinformatics.

In summary, this review studied recent or popular tree visualization tools that can be applied to a wide range of data. The advantages and the disadvantages of each of these tools, as depicted via the heterogeneity of the directions their functionality is focused upon, make them suitable under different circumstances for different applications. The presented characteristics of the mentioned tools are expected to rapidly change due to the continuous improvements both in software and hardware development and do not comprise an exhaustive description of the discussed software's features. In addition, the scope of the descriptions has not been directed towards a comparison and has been restricted in tree associated features, aspects, as well as manipulation and analysis capabilities, only.

Nevertheless, even through the limited notions and perspectives under consideration, an exhaustive overview becomes difficult. This work is focused on recent or popular phylogenetic visualization tools that can be applicable to a wider biological range of data. This part of the survey profiles the functionalities and the application areas of tree visualization tools. Instead of comparing, the survey aims to assist in the selection of the appropriate visualization tool.

The limitations of the tools regarding the manipulation of tree data in biology and their illustration, user-friendliness and interactivity are evident and most of these tools remain limited in terms of usability when thousands of nodes have to be analyzed and visualized. The results of today's large-scale experiments regularly exceed the size of hundred thousands of data points. This issue makes it necessary that more efficient visualization tools need to be developed which are capable of handling, visualizing, processing and analyzing these large datasets. Regarding the layout, more efficient algorithms should be incorporated in order to overcome the current limitations. One promising alternative, accommodated by the modern hardware and software advancements, could be the utilization of 3D space for the representation, as well as the exploration, of trees. This extra dimension can allow a more clear structure and a less cluttered field of view to facilitate smoother and easier navigation within the tree. In addition, extension of the layout algorithms in three dimensions could further render the representation of large-scale networks in a more efficient manner. As depicted in Tables 3 and 4 only few such efforts have taken place today.

To increase the performance of visualization tools further, more efficient handling and allocation of memory will be essential. This can be achieved by loading only the necessary parts of the graph into memory and would multiply the amount of data and taxonomy that can be visualized. Similarly the computational power needed to process and handle very big hierarchies can be split using multiple CPU/processor cores or GPU processors. In such a way re-rooting, deletion or expansion of trees would perform better and in a reasonable amount of time. Visualization tools that will be able to process and analyze huge phylogenetic trees or any kind of hierarchy in real time will become essential in the upcoming years.

The next generation of tree viewers should aim to bridge the gap between analysis and visualization like it already happens with bigger platforms like Matlab, Mathematica, MEGA, the R system or the BioPerl and BioJava modules which are currently poor in visualizations. The aforementioned platforms come with their own tree viewers though those are currently able to give static tree visualizations with no interface interactivity. Statistical, phylogenetic, clustering and mathematical analysis should be incorporated in the newer versions of tree visualizations. Currently scientists and users should be familiar with a variety of platforms and software applications, their advantages and their disadvantages, which often takes precious time and makes research more difficult. In most of the cases users analyze and visualize data independently which many times make it difficult to integrate various software applications especially in cases where they don't follow some of the standard widely used formats.

Finally tree viewers should offer easier annotation of phylogenetic trees bringing information from already existing data sources like Gene Ontology or Mesh terms and offer comparative analysis with protein families, clusters of orthologous groups COGS or existing available trees of life in case of evolution studies. This will make the explorations of data easier and simultaneously help researchers to answer the underlying biological questions more effectively.

In conclusion, future tree visualization tools for life sciences are facing some challenges in the future. The main areas of improvement will be the scalability, the user friendliness and interactivity and the exploitation of a virtual 3D space using modern hardware technologies like multi-core CPU's and GPU's.

## Competing interests

The authors declare that they have no competing interests.

## Authors' contributions

RS is the scientific supervisor of this article. GAP collected the information and wrote the article. TGS and ABS classified and presented the information using tables and wrote parts of this article. All authors read and approved the final manuscript.

## References

[B1] Ashburner M Ball CA Blake JA Botstein D Butler H Cherry JM Davis AP Dolinski K Dwight SS Eppig JT Gene ontology: tool for the unification of biology. The Gene Ontology Consortium Nat Genet 2000 25 1 25 29 10.1038/75556 10802651 PMC3037419

[B2] Bodenreider O The Unified Medical Language System (UMLS): integrating biomedical terminology Nucleic Acids Res 2004 32 Database D267 270 10.1093/nar/gkh061 14681409 PMC308795

[B3] Darwin C The Origin of Species The Modern Library, New York 1872 6 170 171

[B4] Pennisi E Modernizing the tree of life Science 2003 300 5626 1692 1697 10.1126/science.300.5626.1692 12805532

[B5] Jain AK Murty MN Flynn PJ Data Clustering: A review ACM Comp Surv 1999

[B6] Bederson BB Shneiderman B Wattenberg M Ordered and Quantum Treemaps: Making Effective Use of 2D Space to Display Hierarchies ACM Transactions on Graphics (TOG) 2002 21 4 833 854 10.1145/571647.571649

[B7] Johnson B Shneiderman B Treemaps: a space-filling approach to the visualization of hierarchical information structure Proceedings of the second International IEEE Visualization Conference 1991 284 291

[B8] Shneiderman B Tree visualization with tree-maps: A 2-d space-filling approach ACM Transactions on Graphics 1992 11 92 99 10.1145/102377.115768

[B9] James Archie WHED Maddison Wayne Meacham Christopher Rohlf F James Swofford David Felsenstein Joseph The Newick Standard 1986

[B10] Maddison DR Swofford DL Maddison WP NEXUS: an extensible file format for systematic information Syst Biol 1997 46 4 590 621 11975335 10.1093/sysbio/46.4.590

[B11] Swofford DLpaup phylogenetic analysis using parcimony, version 4.0b10 2002 Sinauer Associates, Sunderland, MA12504223

[B12] Ronquist F Huelsenbeck JP MrBayes 3: Bayesian phylogenetic inference under mixed models Bioinformatics 2003 19 12 1572 1574 10.1093/bioinformatics/btg180 12912839

[B13] Felsenstein PHYLIP - Phylogeny Inference Package Cladistics 1989 5 164 166

[B14] Letunic I Bork P Interactive Tree Of Life (iTOL): an online tool for phylogenetic tree display and annotation Bioinformatics 2007 23 1 127 128 10.1093/bioinformatics/btl529 17050570

[B15] Schmidt HA Strimmer K Vingron M Haeseler aAv TREE-PUZZLE: maximum likelihood phylogenetic analysis using quartets and parallel computing Bioinformatics 2002 18 502 504 10.1093/bioinformatics/18.3.502 11934758

[B16] The Newick tree format http://evolution.genetics.washington.edu/phylip/newicktree.html

[B17] Stajich JE Block D Boulez K Brenner SE Chervitz SA Dagdigian C Fuellen G Gilbert JG Korf I Lapp H The Bioperl toolkit: Perl modules for the life sciences Genome Res 2002 12 10 1611 1618 10.1101/gr.361602 12368254 PMC187536

[B18] Holland RC Down TA Pocock M Prlic A Huen D James K Foisy S Drager A Yates A Heuer M BioJava: an open-source framework for bioinformatics Bioinformatics 2008 24 18 2096 2097 10.1093/bioinformatics/btn397 18689808 PMC2530884

[B19] Saitou N Nei M The neighbor-joining method: a new method for reconstructing phylogenetic trees Mol Biol Evol 1987 4 4 406 425 3447015 10.1093/oxfordjournals.molbev.a040454

[B20] Sneath PHA Sokal RR Unweighted Pair Group Method with Arithmetic Mean Numerical Taxonomy 1973 San Francisco: Freeman 230 234

[B21] Ramesh R Chetty M MCMC Based Bayesian Inference for Modeling Gene Networks 2009 vol. 5780/2009, Pattern Recognition in Bioinformatics edn: Springer Berlin/Heidelberg

[B22] Smith TF Waterman MS Identification of common molecular subsequences J Mol Biol 1981 147 1 195 197 10.1016/0022-2836(81)90087-5 7265238

[B23] Thompson JD Higgins DG Gibson TJ CLUSTAL W: improving the sensitivity of progressive multiple sequence alignment through sequence weighting, position-specific gap penalties and weight matrix choice Nucleic Acids Res 1994 22 22 4673 4680 10.1093/nar/22.22.4673 7984417 PMC308517

[B24] Edgar RC MUSCLE: multiple sequence alignment with high accuracy and high throughput Nucleic Acids Res 2004 32 5 1792 1797 10.1093/nar/gkh340 15034147 PMC390337

[B25] Edgar RC MUSCLE: a multiple sequence alignment method with reduced time and space complexity BMC Bioinformatics 2004 5 113 10.1186/1471-2105-5-113 15318951 PMC517706

[B26] Altschul SF Gish W Miller W Myers EW Lipman DJ Basic local alignment search tool J Mol Biol 1990 215 3 403 410 2231712 10.1016/S0022-2836(05)80360-2

[B27] Notredame C Higgins DG Heringa J T-Coffee: A novel method for fast and accurate multiple sequence alignment J Mol Biol 2000 302 1 205 217 10.1006/jmbi.2000.4042 10964570

[B28] Needleman SB Wunsch CD A general method applicable to the search for similarities in the amino acid sequence of two proteins J Mol Biol 1970 48 3 443 453 10.1016/0022-2836(70)90057-4 5420325

[B29] Margulies EH Birney E Approaches to comparative sequence analysis: towards a functional view of vertebrate genomes Nat Rev Genet 2008 9 4 303 313 10.1038/nrg2185 18347593

[B30] Notredame C Recent evolutions of multiple sequence alignment algorithms PLoS Comput Biol 2007 3 8 e123 10.1371/journal.pcbi.0030123 17784778 PMC1963500

[B31] Kemena C Notredame C Upcoming challenges for multiple sequence alignment methods in the high-throughput era Bioinformatics 2009 25 19 2455 2465 10.1093/bioinformatics/btp452 19648142 PMC2752613

[B32] Wilbur WJ Lipman DJ Rapid similarity searches of nucleic acid and protein data banks Proc Natl Acad Sci USA 1983 80 3 726 730 10.1073/pnas.80.3.726 6572363 PMC393452

[B33] Myers EW Miller W Optimal alignments in linear space Comput Appl Biosci 1988 4 1 11 17 3382986 10.1093/bioinformatics/4.1.11

[B34] Michener CD Sokal RR A Quantitative Approach to a Problem in Classification Evolution 1957 11 2 130 162 10.2307/2406046

[B35] Gascuel O Steel M Neighbor-joining revealed Mol Biol Evol 2006 23 11 1997 2000 10.1093/molbev/msl072 16877499

[B36] D'andrade R U-Statistic Hierarchical Clustering Psychometrika 1978 4 58 67

[B37] Johnson SC Hierarchical Clustering Schemes Psychometrika 1967 2 241 254 10.1007/BF02289588 5234703

[B38] Huson DH Richter DC Rausch C Dezulian T Franz M Rupp R Dendroscope: An interactive viewer for large phylogenetic trees BMC Bioinformatics 2007 8 460 10.1186/1471-2105-8-460 18034891 PMC2216043

[B39] Ciccarelli FD Doerks CvM T Creevey C. J Snel B Bork P Towards automatic reconstruction of a highly resolved tree of life Science 2006 311 1283 1287 10.1126/science.1123061 16513982

[B40] Bingham J Sudarsanam S Visualizing large hierarchical clusters in hyperbolic space Bioinformatics 2000 16 7 660 661 10.1093/bioinformatics/16.7.660 11038340

[B41] Perriere G Gouy M WWW-query: an on-line retrieval system for biological sequence banks Biochimie 1996 78 5 364 369 10.1016/0300-9084(96)84768-7 8905155

[B42] hypergeny http://bioinformatics.psb.ugent.be/hypergeny/home.php

[B43] Archer J Robertson DL CTree: comparison of clusters between phylogenetic trees made easy Bioinformatics 2007 23 21 2952 2953 10.1093/bioinformatics/btm410 17717036

[B44] Jordan GE Piel WH PhyloWidget: web-based visualizations for the tree of life Bioinformatics 2008 24 14 1641 1642 10.1093/bioinformatics/btn235 18487241

[B45] Müller J, K M TREEGRAPH: automated drawing of complex tree figures using an extensible tree description format Molecular Ecology Notes 2004 4 786 788 10.1111/j.1471-8286.2004.00813.x

[B46] Dutheil J Galtier N BAOBAB: a Java editor for large phylogenetic trees Bioinformatics (Oxford, England) 2002 18 6 892 893 10.1093/bioinformatics/18.6.892 12075029

[B47] Trooskens G De Beule D Decouttere F Van Criekinge W Phylogenetic trees: visualizing, customizing and detecting incongruence Bioinformatics 2005 21 19 3801 3802 10.1093/bioinformatics/bti590 16030069

[B48] BioNumerics http://www.applied-maths.com/bionumerics/bionumerics.htm

[B49] Slowinski J Review of the computer program Component Cladistics 1993 9 351 353

[B50] Drummond A Strimmer K PAL: an object-oriented programming library for molecular evolution and phylogenetics Bioinformatics 2001 17 7 662 663 10.1093/bioinformatics/17.7.662 11448888

[B51] Lin CY Lin FK Lin CH Lai LW Hsu HJ Chen SH Hsiung CA POWER: PhylOgenetic WEb Repeater--an integrated and user-optimized framework for biomolecular phylogenetic analysis Nucleic Acids Res 2005 33 Web Server W553 556 10.1093/nar/gki494 15980533 PMC1160254

[B52] Kumar S Tamura K Nei M MEGA3: Integrated software for Molecular Evolutionary Genetics Analysis and sequence alignment Brief Bioinform 2004 5 2 150 163 10.1093/bib/5.2.150 15260895

[B53] Tamura K, J D Nei M, S K MEGA4: Molecular Evolutionary Genetics Analysis (MEGA) software version 4.0 Molecular Biology and Evolution 2007 24 1596 1599 10.1093/molbev/msm092 17488738

[B54] Kumar S Tamura K Jakobsen I Nei M MEGA2: molecular evolutionary genetics analysis software Bioinformatics 2001 17 12 1244 1245 10.1093/bioinformatics/17.12.1244 11751241

[B55] Kumar S Tamura K Nei M MEGA: Molecular Evolutionary Genetics Analysis software for microcomputers Comput Appl Biosci 1994 10 2 189 191 8019868 10.1093/bioinformatics/10.2.189

[B56] Maddison DRMWP Mesquite: a modular system for evolutionary analysis 2005 1

[B57] Maddison DRaWPM MacClade version 4: Analysis of phylogeny and character evolution 2000 Sinauer Associates, Sunderland Massachusetts

[B58] Gu S Anderson I Kunin V Cipriano M Minovitsky S Weber G Amenta N Hamann B Dubchak I TreeQ-VISTA: an interactive tree visualization tool with functional annotation query capabilities Bioinformatics 2007 23 6 764 766 10.1093/bioinformatics/btl643 17234642

[B59] Saitou NM The neighbor-joining method: a new method for reconstructing phylogenetic trees Mol Biol Evol 1987 4 4 406 425 3447015 10.1093/oxfordjournals.molbev.a040454

[B60] Desper R Gascuel O Theoretical foundation of the balanced minimum evolution method of phylogenetic inference and its relationship to weighted least-squares tree fitting Mol Biol Evol 2004 21 3 587 598 10.1093/molbev/msh049 14694080

[B61] Rzhetsky A Nei M Theoretical foundation of the minimum-evolution method of phylogenetic inference Mol Biol Evol 1993 10 5 1073 1095 8412650 10.1093/oxfordjournals.molbev.a040056

[B62] Bremer K Branch support and tree stability Cladistics 10 295 304 10.1111/j.1096-0031.1994.tb00179.x

[B63] Efron B Bootstrap Methods: Another Look at the Jackknife The Annals of Statisctics 1979 7 1 1 26 10.1214/aos/1176344552

[B64] Seo J Shneiderman B Interactively Exploring Hierarchical Clustering Results Computer 2002 35 7 80 86 10.1109/MC.2002.1016905

[B65] Seo J Gordish-Dressman H Hoffman EP An interactive power analysis tool for microarray hypothesis testing and generation Bioinformatics 2006 22 7 808 814 10.1093/bioinformatics/btk052 16418236

[B66] Saeed AI Sharov V White J Li J Liang W Bhagabati N Braisted J Klapa M Currier T Thiagarajan M TM4: a free, open-source system for microarray data management and analysis Biotechniques 2003 34 2 374 378 12613259 10.2144/03342mt01

[B67] Pavlopoulos GA O'Donoghue SI Satagopam VP Soldatos TG Pafilis E Schneider R Arena3D: visualization of biological networks in 3D BMC Syst Biol 2008 2 104 10.1186/1752-0509-2-104 19040715 PMC2637860

[B68] Sanderson MJ Paloverde: an OpenGL 3D phylogeny browser Bioinformatics 2006 22 8 1004 1006 10.1093/bioinformatics/btl044 16500938

[B69] Munzner T H3: Laying Out Large Directed Graphs in 3D Hyperbolic Space 1997 IEEE Symposium on Information Visualization. Phoenix, AZ 1997

[B70] Munzner T Exploring Large Graphs in 3D Hyperbolic Space IEEE Computer Graphics and Applications 1998 18 4 18 23 10.1109/38.689657

[B71] Marco A Marin I A general strategy to determine the congruence between a hierarchical and a non-hierarchical classification BMC Bioinformatics 2007 8 442 10.1186/1471-2105-8-442 18005402 PMC2213689

[B72] TreeMe© A software for visualization, manipulation, layouting and labelling of phylogenetic trees http://www.sequentix.de

[B73] Kim N Lee C Three-Dimensional Phylogeny Explorer: distinguishing paralogs, lateral transfer, and violation of "molecular clock" assumption with 3D visualization BMC Bioinformatics 2007 8 213 10.1186/1471-2105-8-213 17584922 PMC1906840

[B74] Nye TM Lio P Gilks WR A novel algorithm and web-based tool for comparing two alternative phylogenetic trees Bioinformatics (Oxford, England) 2006 22 1 117 119 10.1093/bioinformatics/bti720 16234319

[B75] Parr CS Lee B Campbell D Bederson BB Visualizations for taxonomic and phylogenetic trees Bioinformatics 2004 20 17 2997 3004 10.1093/bioinformatics/bth345 15180933

[B76] Rambaut Andrew Charleston M TreeEdit 2002

[B77] Chevenet F Brun C Banuls AL Jacq B Christen R TreeDyn: towards dynamic graphics and annotations for analyses of trees BMC Bioinformatics 2006 7 439 10.1186/1471-2105-7-439 17032440 PMC1615880

[B78] Mavric: a python toolkit for phylogenetics http://www.bioinformatics.org/mavric/

[B79] Paradis E Claude J Strimmer K APE: Analyses of Phylogenetics and Evolution in R language Bioinformatics 2004 20 2 289 290 10.1093/bioinformatics/btg412 14734327

[B80] Hanekamp K Bohnebeck U Beszteri B Valentin K PhyloGena--a user-friendly system for automated phylogenetic annotation of unknown sequences Bioinformatics 2007 23 7 793 801 10.1093/bioinformatics/btm016 17332025

[B81] Carreras M Gianti E Sartori L Plyte SE Isacchi A Bosotti R PoInTree: a polar and interactive phylogenetic tree Genomics Proteomics Bioinformatics 2005 3 1 58 60 16144524 10.1016/S1672-0229(05)03009-3 PMC5172537

[B82] Pasquier C Girardot F Jevardat de Fombelle K Christen R THEA: ontology-driven analysis of microarray data Bioinformatics 2004 20 16 2636 2643 10.1093/bioinformatics/bth295 15130932

[B83] Treemap http://www.cs.umd.edu/hcil/treemap/

